# Plasma Erythropoietin, IL-17A, and IFNγ as Potential Biomarkers of Motor Function Recovery in a Canine Model of Spinal Cord Injury

**DOI:** 10.1007/s12031-020-01575-y

**Published:** 2020-05-16

**Authors:** Lijian Zhang, Xiaoqing Zhuang, Yao Chen, Zhanfeng Niu, Hechun Xia

**Affiliations:** 1 School of Clincial Medicine, Ningxia Medical University, Yinchuan, Ningxia China; 2 Department of Neurosurgery, General Hospital of Ningxia Medical University, No. 804, Shengli Street, Xingqing District, Yinchuan, Ningxia China; 3 Ningxia Human Stem Cell Research Institute, General Hospital of Ningxia Medical University, Yinchuan, Ningxia China; 4 Department of Nuclear Medicine, General Hospital of Ningxia Medical University, No. 804, Shengli Street, Xingqing District, Yinchuan, Ningxia China

**Keywords:** Spinal cord injury, Plasma, Inflammatory cytokines, Motor function, Biomarkers

## Abstract

Traumatic spinal cord injury (SCI) is a devastating neurological disease for which an accurate, cost-effective prediction of motor function recovery is in pressing need. A plethora of neurochemical changes involved in the pathophysiological process of SCI may serve as a new source of biomarkers for patient outcomes. Five dogs were included in this study. We characterized the plasma cytokine profiles in acute phase (0, 1, and 3 days after SCI) and subacute phase (7, 14, and 21 days after SCI) with microarray analysis. The motor function recovery following SCI was monitored by Olby scores. The expression level of differentially expressed proteins (DEPs) was measured with enzyme-linked immunosorbent assay (ELISA). Then, correlations with the Olby scores and receiver operating characteristic curve (ROC) analysis were performed. We identified 12 DEPs including 10 pro-inflammatory and 2 anti-inflammatory cytokines during the 21-day study period. Among those, the expression levels of erythropoietin (EPO), IL-17A, and IFNγ significantly correlated with the Olby scores with *R*^2^ values of 0.870, 0.740, and 0.616, respectively. The results of the ROC analysis suggested that plasma EPO, IL-17A, and IFNγ exhibited a significant predictive power with an area under the curve (AUC) of 0.656, 0.848, and 0.800 for EPO, IL-17A, and IFNγ, respectively. Our results provide a longitudinal description of the changes in plasma cytokine expression in the acute and subacute stages of canine SCI. These data reveal novel panels of inflammation-related cytokines which have the potential to be evaluated as biomarkers for predicting motor function prognosis after SCI.

**Electronic supplementary material:**

The online version of this article (10.1007/s12031-020-01575-y) contains supplementary material, which is available to authorized users.

## Introduction

Traumatic spinal cord injury (SCI) typically results in partial or substantial loss of motor function below the level of injury site, which decreases the quality of life of individuals with SCI (Kumar et al. 2018). Approximately 8000 thousand people will suffer this catastrophic injury each year throughout the world (Gerasimenko et al. 2015). Recently, substantial evidences have shown that the initial neurological examination is extremely difficult for predicting long-term outcomes (Rodrigues et al. 2018; Rasmussen and Carlsen 2016). Even though the injury may seem the same in type and severity, patients could have heterogeneous recovery and uncertain prognosis (Anderson 2004; Raineteau and Schwab 2001). Obtaining accurate and reliable prediction of motor functional improvement is crucial for neurosurgeons and rehabilitation teams to optimize the individualized neuro-restorative treatment options. Therefore, identification of valuable biomarkers of SCI remains a worthy task.

Neurochemical biomarkers have long held promise in the field of SCI to monitor the progression of its pathology and to predict treatment responses longitudinally (Hulme et al. 2017). These biomarkers in general represent molecules released from damaged cells and the acute inflammatory response (acute biomarkers), molecules released during the cellular and immunological response while healing the injury (subacute biomarkers) (Kwon et al. 2019). As peripheral blood is easily accessible and requires less invasive procedures, it was widely used in both animal studies and clinical trials for biomarker screening (Wang et al. 2019). Such protein markers, including tumor necrosis factor-α (TNF-α) and interleukins (ILs), were identified in previous studies (Yousefifard et al. 2019). To date, the vast majority of studies that aimed to discovery plasma/serum biomarkers for SCI have been carried out in preclinical rodent models. However, some hypothesis-driven clinical trials based on rodent results have disappointingly failed, which might be explained by the potential differences between humans and experimental animals (Kwon et al. 2010). Before translating promising findings obtained from the laboratories into clinical settings, preclinical validation of the efficacy in large-animal models is needed. Dogs serve as a good intermediate biological model, bridging the large gap between human and rat studies, as dogs not only have heterogeneity of both injury and genetic backgrounds but also have histopathologic similarities to those in human patients (Dalgaard 2015; Jeffery et al. 2005).

Systemic inflammation plays a pivotal role in promoting many of the pathological consequences of SCI. Previous studies have demonstrated that inflammation-related responses may be a feasible indicator for predicting the prognosis and severity of SCI (Yang et al. 2005; Kijima et al. 2019). Therefore, a greater insight into the molecular dynamics underlying the neuroinflammation process could enable us to develop novel better biomarkers. In this longitudinal study, we aimed to characterize the inflammation-related cytokine profiles in the acute and subacute stage of canine SCI and identify new peripherally accessible biomarkers which may have the potential to be used for SCI prognostication.

## Methods and Materials

### Establishment of SCI Dog Model

A total of 5 healthy vaccinated 12–24-month-old beagle dogs weighing 13–17 kg were used in this study. All surgical procedures followed the standards and guidelines set out by the Experimental Animal Centre of Ningxia Medical University (2017-073). The painkiller (tramadol hydrochloride, CSPC Pharmaceutical Co., Ltd., Shijiazhuang, China; 2.5 mg/kg, intramuscularly) was given to the dogs 30 min before the operation. The operation was carried out under general anesthesia with 2.0–3.0% isoflurane in oxygen. During the operation, body temperature was maintained using a heating pad. Using sanitized instruments, laminectomy was performed at the tenth thoracic segment (T10), followed by a unilateral left side hemisection at the T10 level, which was performed by micro-scissors. Before voluntary urination establishment, manual bladder expression was performed at least three times a day.

### Protein Antibody Array

Plasma samples (5 ml) were collected from the jugular vein of each dog for cytokine analysis at 0 day (pre-SCI), 1 day, 3 days, 7 days, 14 days, and 21 days post-injury (dpi). According to the manufacturer’s instructions, plasma samples and standard curve cocktails were incubated on a Quantibody Canine Cytokine Array Q1/2/3/4 (RayBiotech, Norcross, GA, USA), which simultaneously detects 40 cytokines (details in Supplementary Table S1). After the experimental procedures, the glass slides were scanned to detect the fluorescent signals in the microarray, and the signals were visualized using a GenePix Professional 4200A scanner (Molecular Devices, Sunnyvale, CA, USA). Data extraction was performed using GenePix Pro 5.1 software. Cytokine levels were normalized to milligrams of tissue.

### Cytokine Measurement with Enzyme-Linked Immunosorbent Assay

The plasma levels of EPO, IL-17A, and IFNγ were measured by enzyme-linked immunosorbent assay (ELISA) (RayBiotech, Norcross, GA, USA) according to the manufacturer’s instructions. In brief, protein extracts of the plasma samples were incubated in plates coated with the capture antibody overnight at 4 °C. The plates were washed, and a biotin-conjugated detection antibody was added to the plates and incubated at room temperature for 2 h to bind to the corresponding proteins. HRP-conjugated streptavidin was added to the plates and allowed to incubate for 45 min. Tetramethylbenzidine dihydrochloride (TMB) reagent was added and allowed to incubate for 30 min before the reaction was stopped with sulfuric acid. The optical density was measured via an ELx800NB microplate reader (BioTek, Winooski, CT, USA) at a wavelength of 450 nm.

### Motor Function Recovery Assessment

All dogs were subjected to motor function assessment to exclude any motor deficits before the experiment. After the establishment of SCI model, the dogs were observed for any spontaneous recovery of motor function using Olby score (Olby et al. 2001) in the acute phase (0, 1, and 3 dpi) and the subacute phase (7, 14, and 21 dpi) (Moore et al. 2017).

### Statistical Analysis

All data were statistically analyzed by using the GraphPad Prism (version 7.0, GraphPad Software Inc., San Diego, CA, USA) with nonparametric Kruskal-Wallis test followed by Dunnett’s multiple comparison. Correlation analysis was performed using Pearson’s product moment (*R*) correlation. The area under the receiver operating characteristic (ROC) curve was performed for predictive power estimation. All values are presented as the mean ± standard deviation (SD). *p* < 0.05 was considered statistically significant.

## Results

### Cytokine Profiling in Plasma

**Fig. 1:**
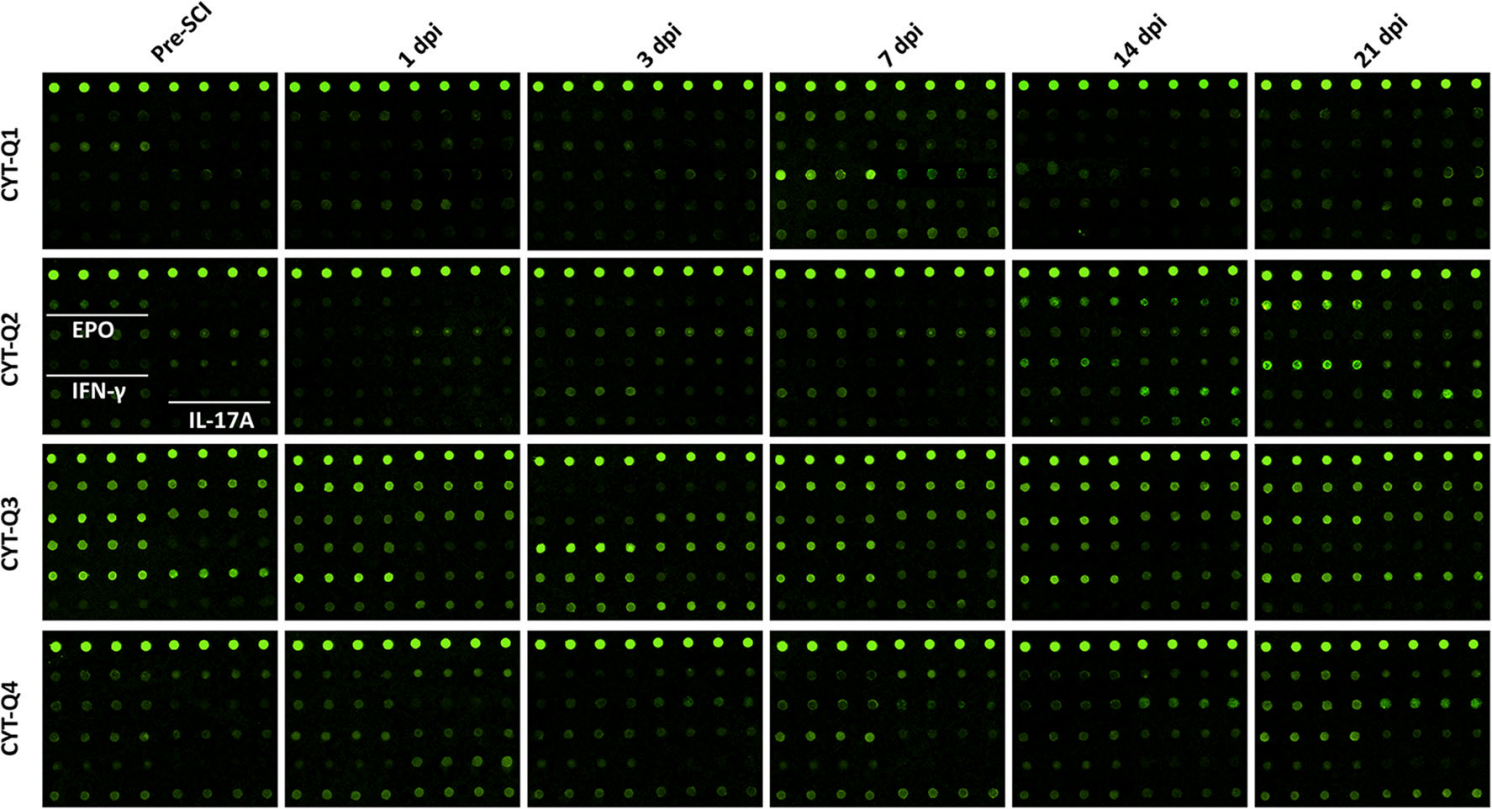
Fluorescence images of microarray chips. The location of EPO, IFNγ, and IL-17A in the array is labeled by white underscores. The fluorescence intensity of the spots indicates the level of expression

**Fig. 2:**
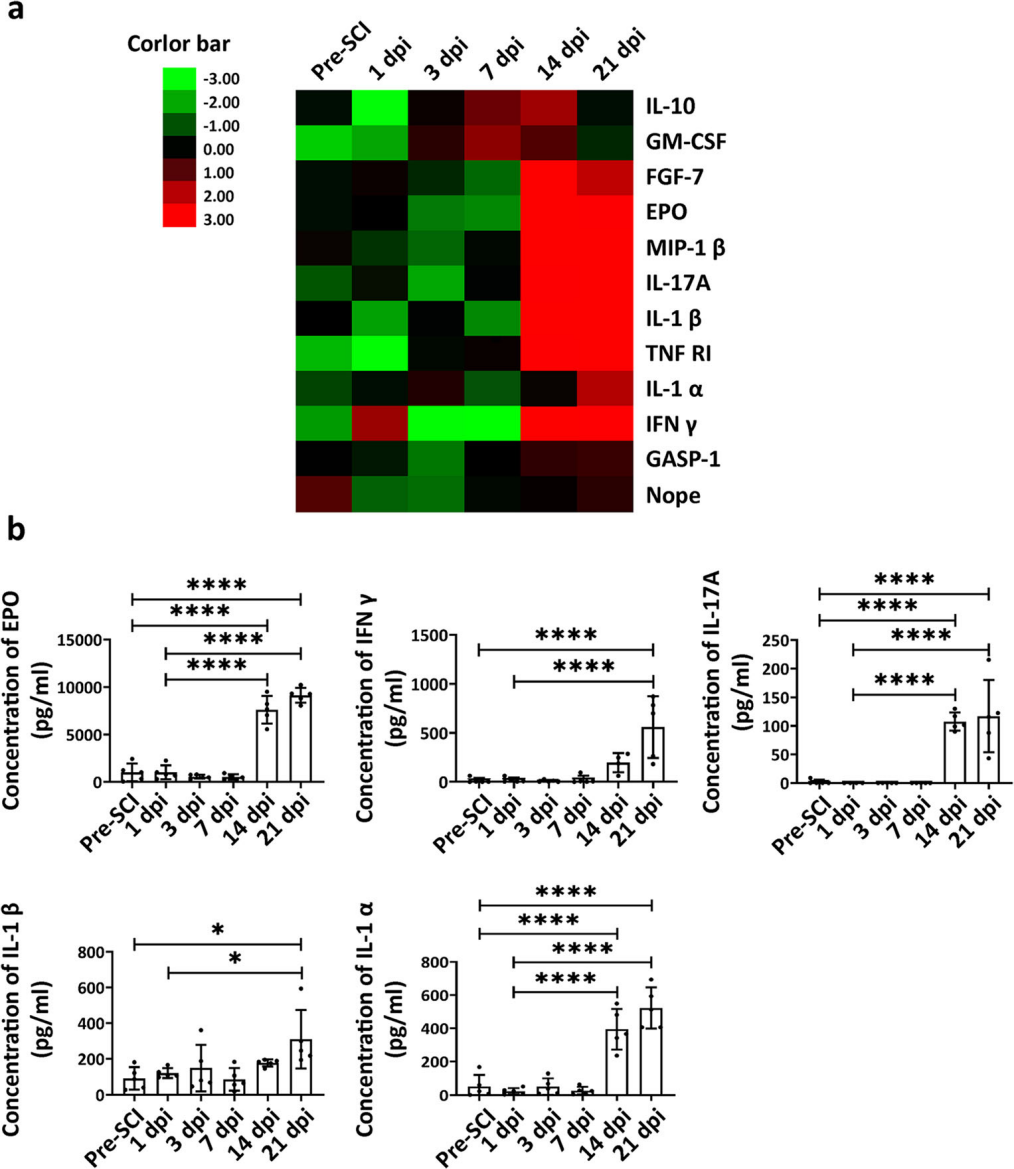
The temporal profile of plasma cytokines following SCI. **a** Heat map analysis of differentially expressed cytokines following SCI in dogs. Color bar: green indicates downregulation; red indicates upregulation. **b** Temporal expression of the selected pro- and anti-inflammatory cytokines in plasma of SCI dogs: EPO, IFNγ, IL-17A, IL-1β, and IL-1α. Data are presented as mean ± SD, *n* = 5 in each group, *p < 0.05, ****p < 0.0001

In this study, we investigate the temporal changes in plasma cytokines obtained from SCI dogs with protein chip array (Fig. 1). Heat map analyses showed significant changes in the concentration of 10 pro-inflammatory cytokines (GM-CSF, GASP-1, FGF7, TNF R1, NOPE, IL-1α, IL-1β, IFNγ, MIP-1β, and IL-17A) and 2 anti-inflammatory cytokines (EPO and IL-10) after SCI (Table 1, Fig. 2a). At 14 and 21 dpi, the concentrations of both IL-17A (*p* < 0.0001, *n* = 5; *p* < 0.0001, *n* = 5) and IFNγ (*p* < 0.0001, *n* = 5; *p* < 0.0001, *n* = 5) were significantly elevated. The level of EPO tended to decrease within 7 dpi, but without significant difference with pre-SCI level, and then increased markedly at 14 and 21 dpi (*p* < 0.0001, *n* = 5; *p* < 0.0001, *n* = 5, respectively). And the concentration of IL-1α fluctuated within first 7 dpi with an initially decreased expression on 1 dpi (*p* < 0.05, *n* = 5) that then became significantly elevated on 14 and 21 dpi (*p* < 0.0001, *n* = 5; *p* < 0.0001, *n* = 5). However, the expression of IL-1β appeared to be biphasic, with two peaks at 3 dpi and 21 dpi, and the significant changes occurred at 21 dpi (*p* = 0.40, *n* = 5; *p* < 0.0001, *n* = 5, respectively) (Fig. 2b).

**Table 1:** The list of differentially expressed cytokines (pg/ml)

	Pre-SCI	1 dpi	3 dpi	7 dpi	14 dpi	21 dpi
IL-10	74.04 ± 41.92	20.67 ± 19.14****	83.66 ± 34.80	130.09 ± 49.57	176.79 ± 120.53	74.38 ± 40.19
EPO	1011.78 ± 830.46	1009.3 ± 666.14	552.78 ± 188.21	522.78 ± 250.49	7613.48 ± 1308.58****	9146.79 ± 700.23****
MIP 1β	65.39 ± 60.59	49.29 ± 30.43	38.55 ± 26.19	59.96 ± 22.60	849.71 ± 109.22****	1062.1 ± 207.89****
GM-CSF	9.41 ± 6.77	11.36 ± 14.67	30.89 ± 14.11	50.40 ± 16.31**	37.38 ± 16.83	21.10 ± 11.84
GASP 1	2082.21 ± 564.18	1834.3 ± 224.79	1202.59 ± 266.79*	2080.71 ± 639.20	2582.31 ± 186.45	2692.17 ± 282.30
FGF7	38.87 ± 48.56	43.87 ± 59.20	34.84 ± 20.52	25.24 ± 11.72	153.34 ± 70.31*	105.40 ± 50.63
TNF R1	4.55 ± 9.10	1.79 ± 3.57	10.61 ± 18.01	11.53 ± 23.06	279.21 ± 163.72***	197.44 ± 108.16*
NOPE	4242.31 ± 1871.29	1763.04 ± 628.61	1652.22 ± 1186.37*	2735.73 ± 839.79	2938.60 ± 1039.98	3439.99 ± 963.74
IFNγ	1.76 ± 3.52	38.29 ± 15.31	0.00 ± 0.00	0.00 ± 0.00	537.70 ± 14.19****	584.86 ± 56.55****
IL-1α	52.01 ± 61.16	23.54 ± 17.05	51.48 ± 44.15	26.81 ± 20.17	394.88 ± 109.51****	522.24 ± 110.69****
IL-1β	91.82 ± 55.51	121.18 ± 23.85	149.08 ± 116.42	86.20 ± 56.11	177.42 ± 17.38	310.04 ± 146.17*
IL-17A	13.32 ± 24.18	18.61 ± 22.19	8.93 ± 10.06	21.65 ± 36.92	394.73 ± 404.11	560.61 ± 282.52**

Compared with baseline expression level of pre-SCI dogs. Data are presented as mean ± SD, *n* = 5 in each group, *p < 0.05, **p < 0.01, ***p < 0.001, ****p < 0.0001

### Assessment of Motor Function

**Fig. 3:**
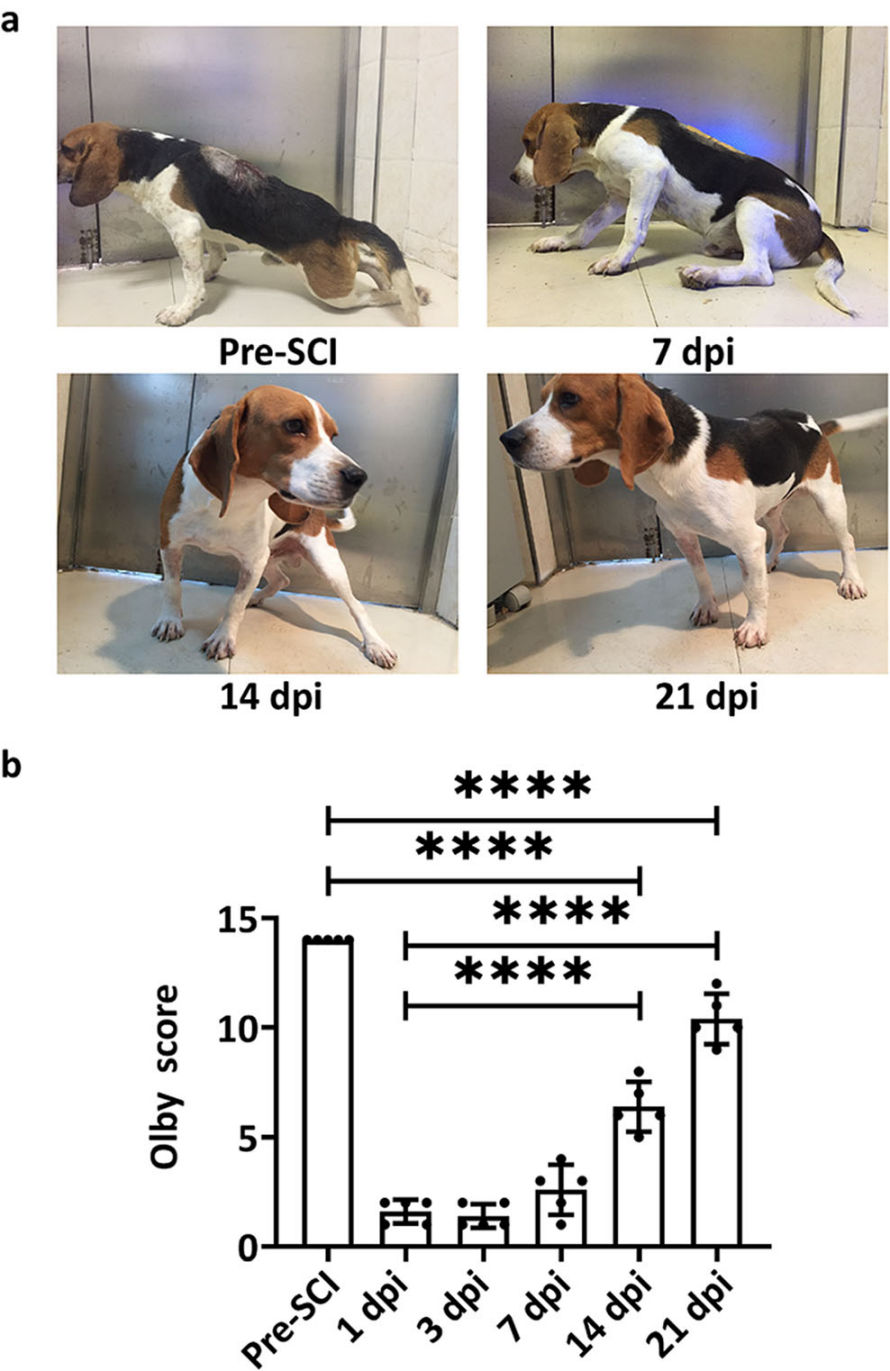
Motor function assessments with Olby score. **a** Representative images of motor functional recovery of SCI dogs. **b** Changes in the Olby scores during the 21-day study period. Data are presented as mean ± SD, *n* = 5 in each group, ****p < 0.0001

The motor function of SCI dogs was assessed using the Olby score. As shown in Fig. 3, all dogs showed an Olby score of 1, suggesting no motor function in the left lower limb after the establishment of SCI. Gradual recovery of motor function in the paralyzed lower limb was observed in all dogs after SCI. The Olby score reached significance at 14 dpi and 21 dpi compared with that at 1 dpi (6.40 ± 1.02 vs 1.60 ± 0.49, *p* < 0.0001, *n* = 5; 10.04 ± 1.01 vs 1.60 ± 0.49, *p* < 0.0001, *n* = 5).

### Plasma EPO, IFNγ, and IL-17A Concentrations for Motor Functional Prognosis

**Fig. 4:**
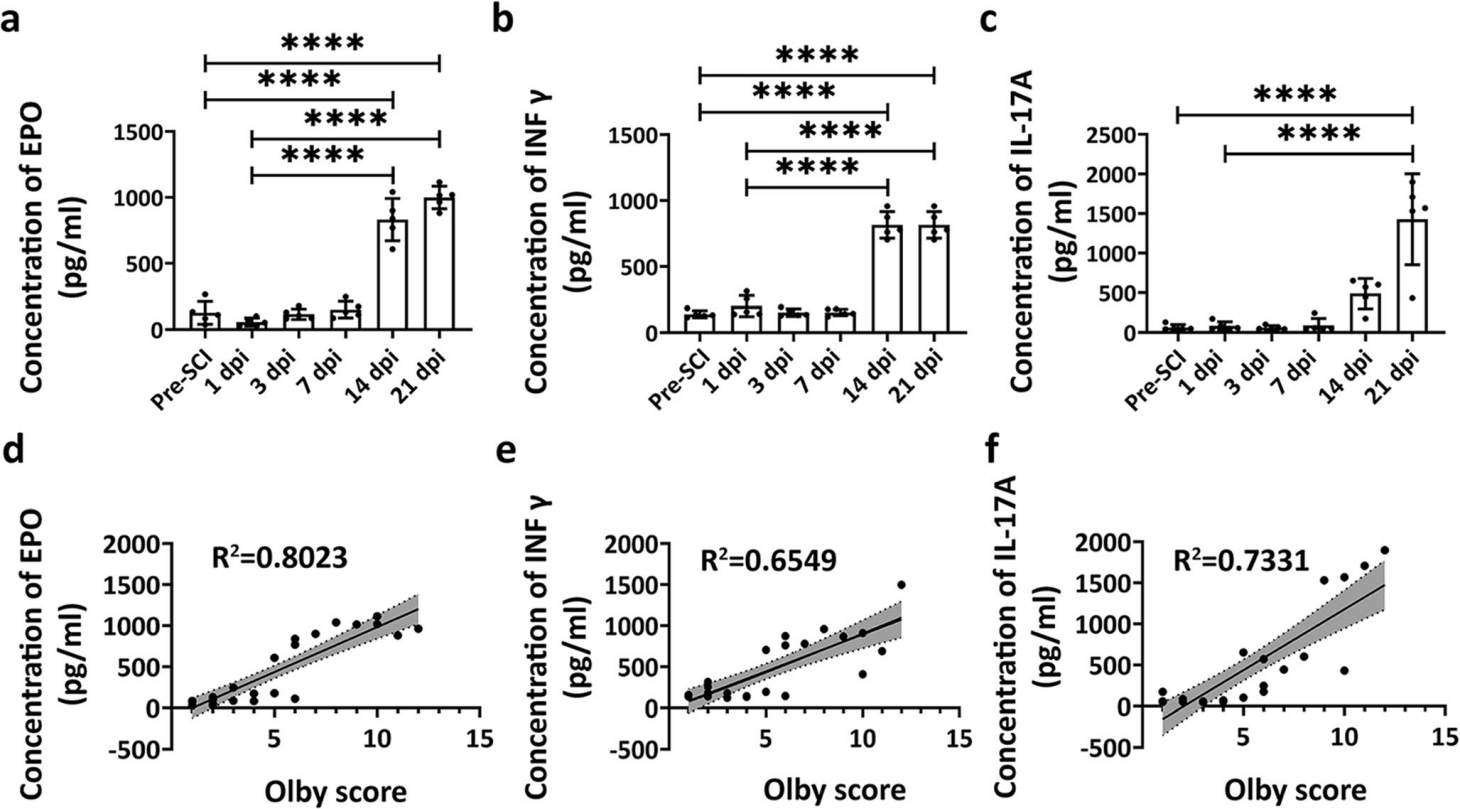
ELISA validation for EPO, IFNγ, and IL-17A and correlation with the Olby score. The concentration of EPO (**a**), IFNγ (**b**), and IL-17A (**c**) obtained from ELISA are shown by scatter plot. Correlation between the Olby score and EPO (**d**), IFNγ (**e**), and IL-17A (**f**) concentrations. Data are presented as mean ± SD, *n* = 5 in each group, ****p < 0.0001

To validate the expression level of EPO, IFNγ, and IL-17A, the ELISA analysis was performed. In accordance with the results obtained from the Quantibody Cytokine Array, the ELISA experiments showed that IL-17A, IFNγ, and EPO were significantly upregulated at 14 and 21 dpi (*p* < 0.0001, *n* = 5; *p* < 0.0001, *n* = 5; and *p* < 0.0001, *n* = 5, respectively) (Fig. 4a–c).

To investigate whether the concentration of EPO, IFNγ, and IL-17A could serve as biomarkers of motor function recovery, we next performed a linear regression analysis. Our results showed that the concentration of EPO (*R*^2^ = 0.8023, *p* < 0.0001), IFNγ (*R*^2^ = 0.6549, *p* < 0.0001), and IL-17A (*R*^2^ = 0.7331, *p* < 0.0001) correlated well with the Olby score (Fig. 4d–f).

### ROC Analysis

**Fig. 5:**
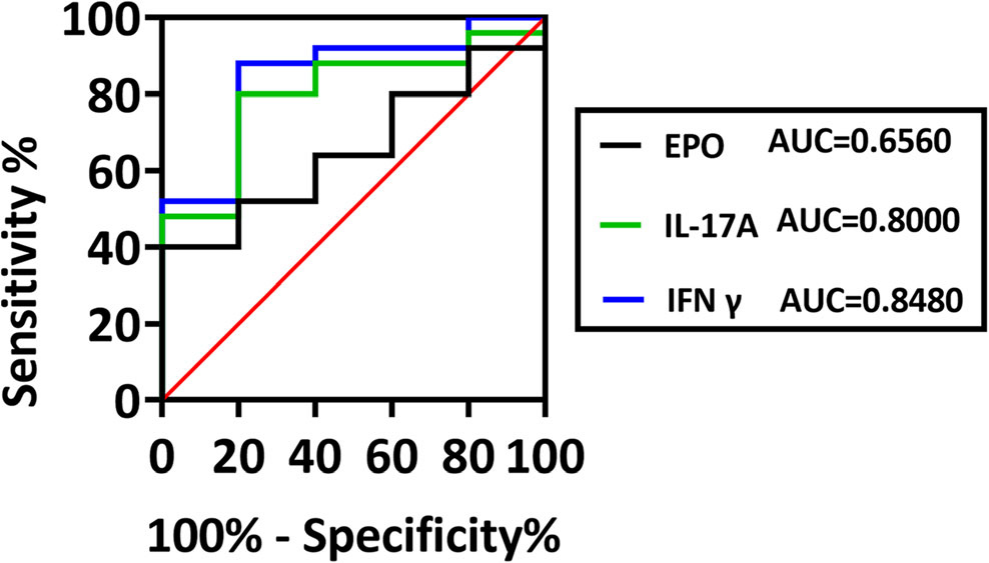
Receiver operating characteristic (ROC) curve analysis. ROC curve analysis of EPO, IL-17A, and IFNγ for determining their prognostic abilities. AUC, area under the ROC curve

The ROC curve of the cytokines reflected separation between SCI dogs and dogs without SCI, with an area under the curve (AUC) of 0.656, 0.848, and 0.800 for EPO, IL-17A, and IFNγ, respectively (Fig. 5). Then, we compared the specificity and sensitivity for each plasma protein individually (Table 2).

**Table 2:** Comparison of the predictive abilities of the selected cytokines

	Sensitivity (%)	Specificity (%)	AUC	95% CI
EPO	40	100	0.656	23.40 to 59.26
IFNγ	88	80	0.848	70.04 to 95.83
IL-17A	80	80	0.800	60.87 to 91.14

*AUC*, area under the ROC curve

## Discussion

To date, several research groups have reported poor feasibility and accuracy of current methods in classifying injury severity and predicting neurologic outcomes due to the heterogeneity of SCI or limitations of each imaging technology (Krishna et al. 2014; Hicks et al. 2017). For example, Kaminski et al. (2017) created a prediction model including four different acute clinical parameters associated with an *R*^2^ value of 0.573. Here, we investigated the temporal changes in the expression level of 40 cytokines using cytokine arrays. Our results showed that inflammation-related cytokines exhibited improved predictive power with *R*^2^ values of 0.870 (EPO), 0.740 (IFNγ), and 0.616 (IL-17A) in acute and subacute stages of SCI. This indicates that plasma EPO, IL-17A, and IFNγ could be potential biomarkers for motor functional prognosis in the acute and subacute phases of SCI.

EPO is a robust biomarker of motor functional recovery identified in our current study. Until now, a substantial number of animal models and clinical trials have shown the efficiency of both endogenous and exogenously administered EPO for neurological recovery (Costa et al. 2015; Carelli et al. 2017; Grasso et al. 2005). Little is known about its potential to serve as a biomarker for SCI. To the best of our knowledge, this is the first study investigating the correlation of endogenous levels of EPO with motor function following SCI. Previous studies have reported that the release of pro-inflammatory cytokines or metabolic stress induced by tissue injury could activate EPO production (Carelli et al. 2011). And EPO could protect a wide variety of cells and tissues in turn, from apoptosis induced by hypoxia, as well as from excitotoxins and glucose deprivation following SCI (Matis and Birbilis 2009; Foley et al. 2017). Evidence has shown that the plasma EPO is significantly increased in SCI patients, returning to normal after 8 weeks (Claus-Walker and Dunn 1984). As a tissue-protective cytokine, the neuroregenerative effects of EPO have been extensively investigated in various species SCI model (Celik et al. 2002; Simon et al. 2011; Simon et al. 2016). EPO reduced astrogliosis (Vitellaro-Zuccarello et al. 2008) and scar formation (Gorio et al. 2005) and enhanced remyelination by promoting oligodendrogenesis (Cho et al. 2012), thus improving functional neurological status after SCI. The findings obtained from Hong et al.’s study also demonstrated that EPO could increase neurite outgrowth after SCI. Spinal cord neurons grown in the absence of EPO were shorter and fewer in number compared with those grown in the presence of EPO (Hong et al. 2018). Such benefits of neuroprotective activity may underlie the correlation of EPO with motor function recovery following SCI, as was observed in this study.

Among the differentially expressed proteins (DEPs), the expression level of IL-17A and IFNγ was also positively correlated with the motor function. Substantial studies suggest that IL-17A is not only exclusively a pro-inflammatory factor but also has a potential role in neuroanatomical plasticity (Moynes et al. 2014; Chisholm et al. 2012; Habash et al. 2015). The results obtained from Hu et al.’s study showed that the high level of IL-17A treatment could protect neurons from apoptosis via the activation of JAK2/STAT-3 signaling and the suppression of voltage-dependent Ca^2+^ influx. And the blockade of IL-17A function at late phases of experimental autoimmune uveoretinitis (EAU) resulted in significantly neural cell apoptosis and tissue damage (Hu et al. 2014). Another study also suggested that the high dose of IL-17A administration exerts anti-apoptotic and neuroprotective activity via miR-155-5p downregulation (Ksiazek-Winiarek et al. 2017). Similarly, IFNγ is also a pleiotropic cytokine that exhibits both detrimental and neuroprotective effects. Increasing evidences have indicated the beneficial aspects of upregulated IFNγ in SCI. Fujiyoshi and his colleagues (Fujiyoshi et al. 2010) showed that the intraperitoneal administration of IFNγ significantly facilitated the recovery of motor function with reduced inflammatory cells concentrated in the injury site following SCI. In addition, SCI applied to IFNγ KO mice resulted in a greater proportion of degenerating neurons in the ventral horn (Victório et al. 2010). IFNγ also can activate the choroid plexus to boost the recruitment of inflammation-resolving cells such as T cells and monocyte-derived macrophages which support functional recovery following SCI (Kunis et al. 2013). Moreover, Ishii et al. (2013) suggested that IFNγ contributes to the upregulation of glial cell line–derived neurotrophic factor (GDNF) and the secretion of IL-10 in Th1 cells, which might have positive effects on neuroprotection after SCI. And the transfer of IFNγ-producing Th1 cells could enhance axonal remodeling of corticospinal tract and serotonergic fibers, and promote the functional recovery in SCI mice (Ishii et al. 2012). In light of the above literatures, we proposed that the improved motor function observed in our study might be related to their neuroprotective effects. Histological examination of spinal cord tissue that reveals the neuroanatomical changes should be included in our future work.

Other identified DEPs, such as IL-1α and IL-1β, which were reported to be potential biomarkers for SCI (Yokobori et al. 2015), did not reach any statistical significance correlation with the motor function. Moreover, the IL-1β detection in blood is in a very limited period of time, because the increased IL-1β can be found mainly during a time when no leucocytosis appears (Christian et al. 2013).

However, this study has limitations. First, the sample size of this study is small and the available protein antibody arrays for canine study are limited. A larger and prospective cohort study with different severity of SCI canines should be conducted to validate our results in the future. Second, this study is only focused on the acute and subacute stages of SCI. It would be also very important to investigate the alternations in the expression level of cytokines and their correlation with motor function in the chronic phase of SCI.

## Conclusion

Our findings provided a comprehensive description of dynamic cytokine profiles in the acute and subacute stages of SCI canines. In this longitudinal study, we identified panels of inflammation-related biomarker candidates including EPO, IFNγ, and IL-17A with high potential to predict motor function prognosis following the acute and subacute phase of SCI.

## Electronic Supplementary Material

ESM 1 (TIF 7782 kb)

High Resolution Image (PNG 429 kb)

ESM 2 (DOCX 18 kb)

## Abbreviations

DEPs: differentially expressed proteins
EPO: erythropoietin
TNF-α: tumor necrosis factor-α
ILs: interleukins
Dpi: days post-injury
AUC: area under the curve
ELISA: enzyme-linked immunosorbent assay
TMB: tetramethylbenzidine dihydrochloride
GO: Gene Ontology
KEGG: Kyoto Encyclopedia of Genes and Genomes
GDNF: glial cell line–derived neurotrophic factor
CST: corticospinal tract
EAU: experimental autoimmune uveoretinitis
