# Supplementary figures and images for: Plasma Erythropoietin, IL-17A, and IFNγ as Potential Biomarkers of Motor Function Recovery in a Canine Model of Spinal Cord Injury
Source: J Mol Neurosci. 2020 May 16;70(11):1821–8. doi: 10.1007/s12031-020-01575-y (PMC7561571; doi:10.1007/s12031-020-01575-y)

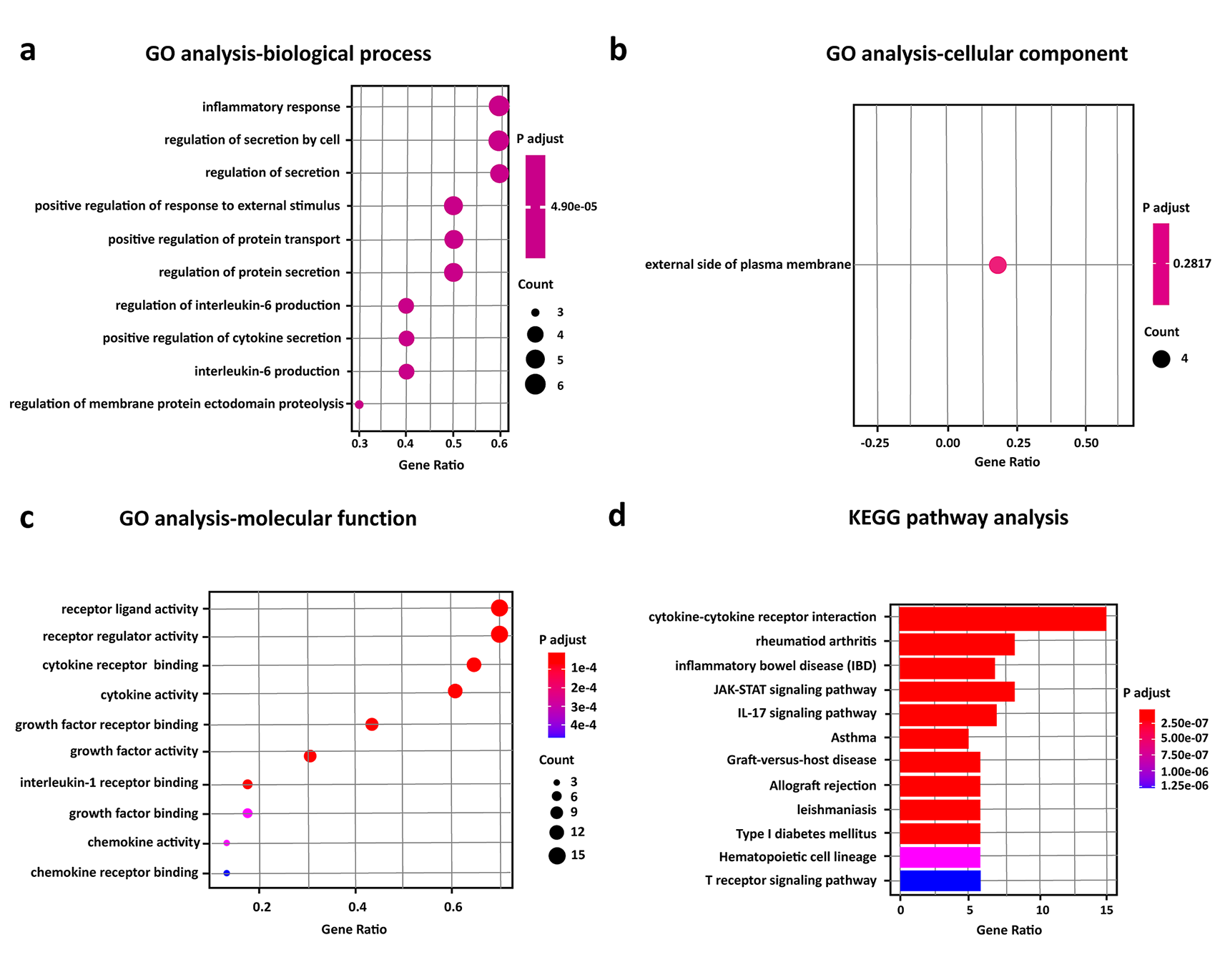

Supplement: Supplementary file 1 — (TIF 7782 kb) [file 12031_2020_1575_MOESM1_ESM.tif]

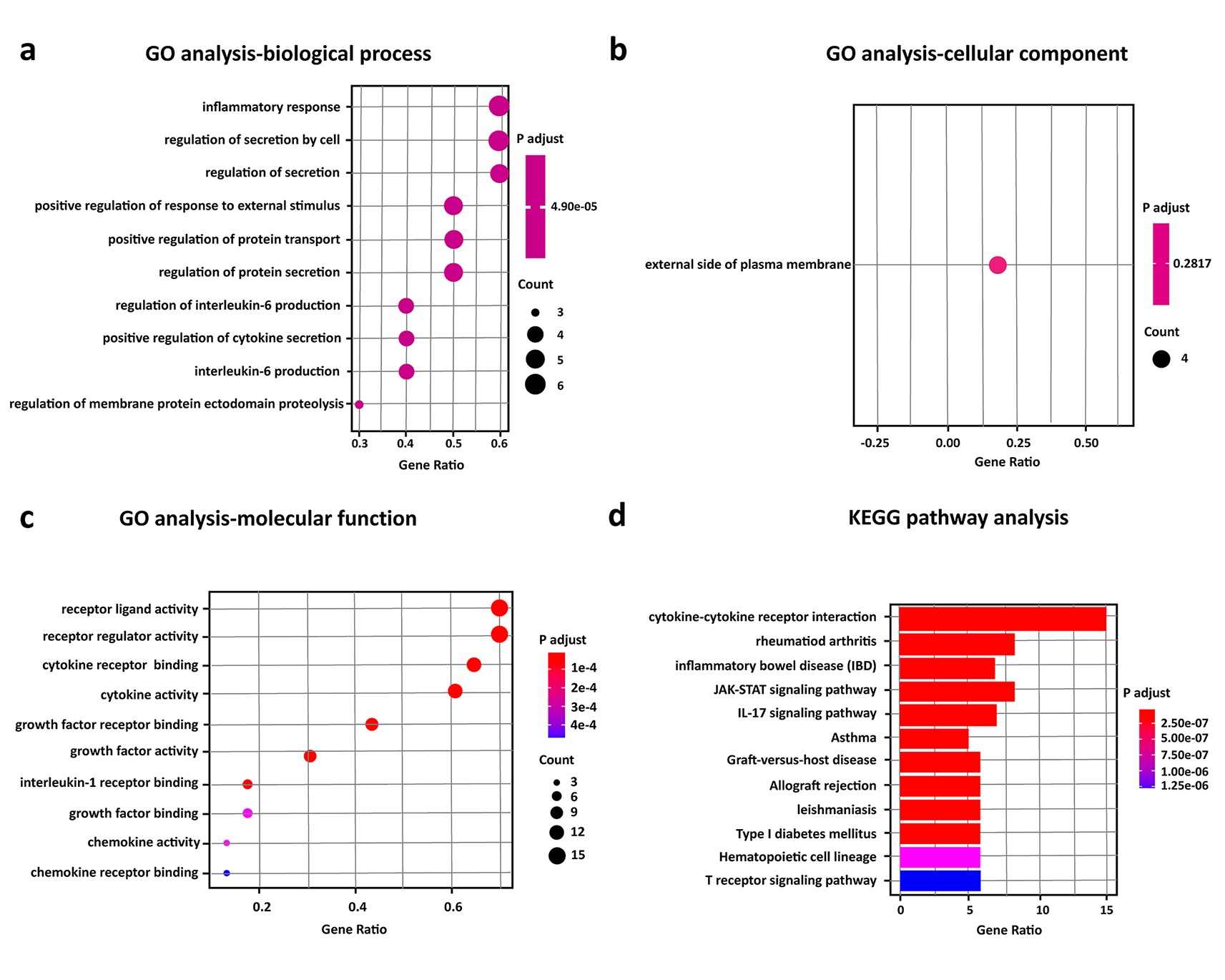

Supplement: Supplementary file 2 — High Resolution Image (PNG 429 kb) [file 12031_2020_1575_Fig6_ESM.png]
