# Supplementary material for: Plasma Erythropoietin, IL-17A, and IFNγ as Potential Biomarkers of Motor Function Recovery in a Canine Model of Spinal Cord Injury
Source: J Mol Neurosci. 2020 May 16;70(11):1821–8. doi: 10.1007/s12031-020-01575-y (PMC7561571; doi:10.1007/s12031-020-01575-y)
Supplement: Supplementary file 3 — (DOCX 18 kb) [file 12031_2020_1575_MOESM2_ESM.docx]

**Supplementary Table S1: Quantibody Canine Cytokine Array Q1/2/3/4, and each antibody is printed in quadruplicate horizontally.**

Canine Cytokine Array Q1 Map

|  | 1 | 2 | 3 | 4 | 1 | 2 | 3 | 4 |
| --- | --- | --- | --- | --- | --- | --- | --- | --- |
| A | POS1 | | | | POS2 | | | |
| B | IL-2 | | | | IL-6 | | | |
| C | IL-8 (CXCL8) | | | | IL-10 | | | |
| D | GM-CSF | | | | MCP-1 (CCL2) | | | |
| E | RAGE | | | | SCF | | | |
| F | TNF-α | | | | VEGF-A | | | |

Canine Cytokine Array Q2 Map

|  | 1 | 2 | 3 | 4 | 1 | 2 | 3 | 4 |
| --- | --- | --- | --- | --- | --- | --- | --- | --- |
| A | POS1 | | | | POS2 | | | |
| B | Erythropoietin | | | | FGF-7 (KGF) | | | |
| C | HGF | | | | HGFR | | | |
| D | IFN γ | | | | IL-1β | | | |
| E | IL-12 P40 | | | | IL-17A | | | |
| F | MIP-1β | | | | TNF-RI (TNFRSF1A) | | | |

Canine Cytokine Array Q3 Map

|  | 1 | 2 | 3 | 4 | 1 | 2 | 3 | 4 |
| --- | --- | --- | --- | --- | --- | --- | --- | --- |
| A | POS1 | | | | POS2 | | | |
| B | ErbB3 (Her3) | | | | Galectin-3 | | | |
| C | GASP-1 | | | | IGFBP-2 | | | |
| D | KC (CXCL1) | | | | MMP-8 | | | |
| E | NCAM-1 (CD56) | | | | Nope | | | |
| F | FDGF-BB | | | | TGF-α | | | |

Canine Cytokine Array Q4 Map

|  | 1 | 2 | 3 | 4 | 1 | 2 | 3 | 4 |
| --- | --- | --- | --- | --- | --- | --- | --- | --- |
| A | POS1 | | | | POS2 | | | |
| B | Cystatin C | | | | Decorin | | | |
| C | EGFR | | | | IL-1α | | | |
| D | IL-1ra | | | | IL-13 | | | |
| E | IL-21 | | | | RANTES (CCL5) | | | |
| F | Resistin | | | | Trappin-2 (Elafin) | | | |
